# Therapeutic potential of ginseng and its bioactive compounds in inflammatory bowel disease: current evidence and future directions

**DOI:** 10.3389/fimmu.2026.1878839

**Published:** 2026-08-10

**Authors:** Md Minarul Islam, Il-Ho Park, Ki Sung Kang

**Affiliations:** 1 Department of Food Science and Biotechnology, Gachon University, Seongnam, Republic of Korea; 2 College of Pharmacy, Sahmyook University, Seoul, Republic of Korea; 3 College of Korean Medicine, Gachon University, Seongnam, Republic of Korea

**Keywords:** cytokines, ginsenosides, immune cells, inflammation, inflammatory bowel diseases, signaling pathways

## Abstract

Inflammatory bowel disease (IBD) is a group of chronic immune-mediated inflammatory disease characterize by persistent inflammation of the gastrointestinal tract that can substantially impact quality of life. Due to the inconsistencies and adverse effects associated with conventional therapies, there is an increasing demand for safer and more reliable treatment alternatives for IBD. The pathogenesis of IBD involves multiple factors, including the overactivation of signaling pathways, elevated pro-inflammatory cytokine levels, dysbiosis of the gut microbiota, and activation of immune cells. Traditionally, ginseng has been used as both a dietary component and a therapeutic agent, its bioactive constituents (ginsenosides and their derivatives) have demonstrated potential therapeutic effects in preclinical IBD models. Numerous studies have shown that ginsenosides may ameliorate experimental colitis through multiple mechanisms, including the inhibition of inflammatory signaling pathways, regulation of cytokine production, modulation of immune cell activity, and alteration of the gut microbiota. However, clinical studies investigating the use of ginsenosides in the treatment of IBD are extremely limited. In this review, we provide a comprehensive overview of IBD pathology and highlight the therapeutic potential of ginseng and its derivatives, particularly ginsenosides, as emerging therapeutic option, along with their current challenges and prospects.

## Graphical Abstract

**Figure:**
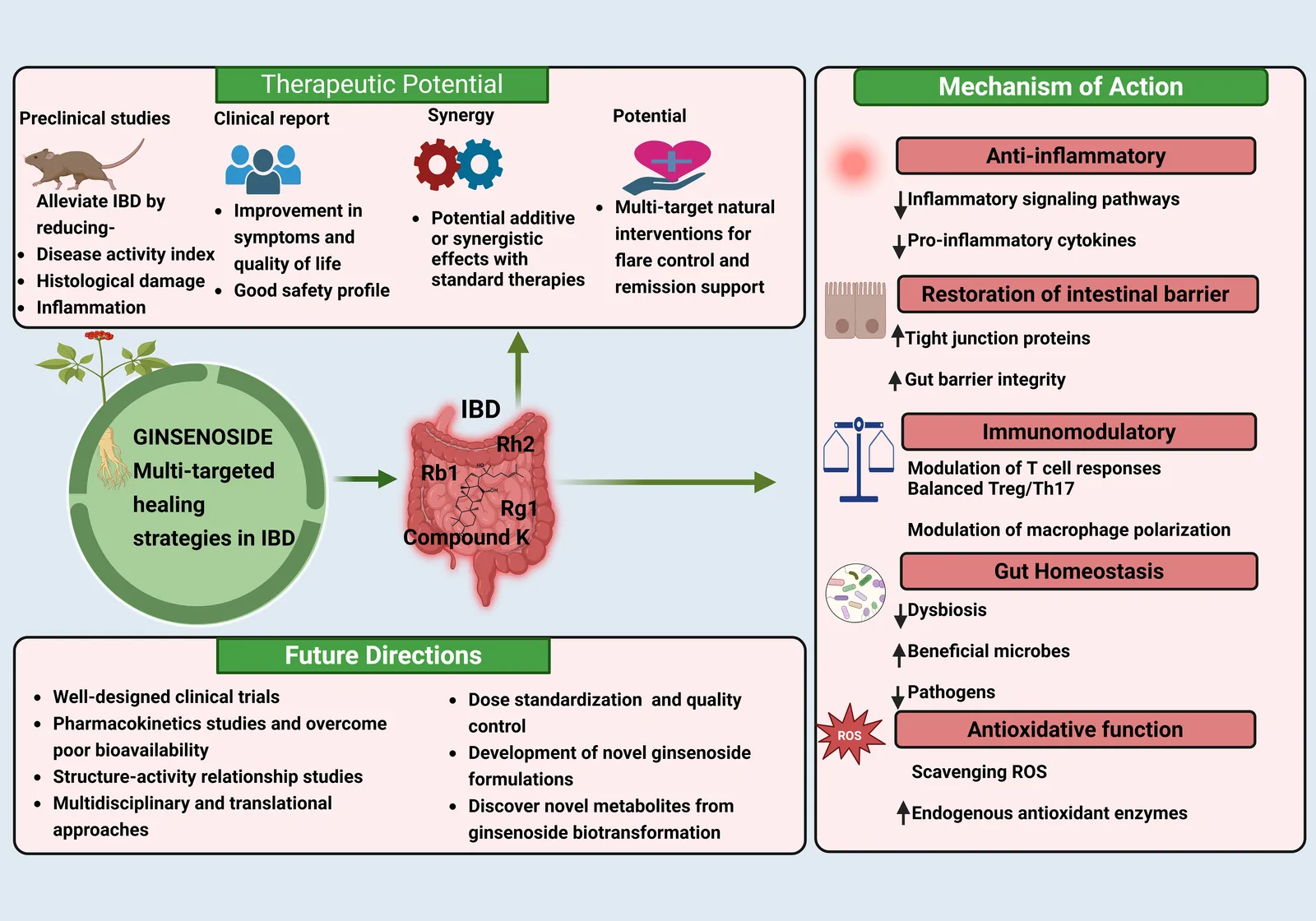

## 1 Introduction

Inflammatory bowel disease (IBD) encompasses ulcerative colitis (UC) and Crohn’s disease (CD), both of which are chronic and recurrent inflammatory conditions of the gastrointestinal tract characterized by persistent intestinal inflammation, disruption of the epithelial barrier, and symptoms such as abdominal pain and bloody diarrhea (1, 2). Although its precise etiology is unknown, IBD is believed to stem from a complex interplay of genetic predisposition, immune system dysfunction, environmental influences, and gut microbiome imbalances. These processes result in heightened intestinal permeability, increased immune cell infiltration, altered T-cell and macrophage activity, and microbial dysbiosis—characterized by decreased short-chain fatty acid (SCFA)–producing bacteria and increased pro-inflammatory microbes—thus perpetuating chronic intestinal inflammation (3, 4). The 2017 Global Burden of Disease (GBD) study estimated that approximately 6.8 million individuals worldwide (95% uncertainty interval: 6.4–7.3 million) were affected by IBD, underscoring its significant global impact (5). Recent GBD data (up to 2023) have shown a continuing global increase in both the incidence and prevalence of IBD, with the age-standardized incidence escalating from 12.3 per 100,000 in 1990 to 25.6 per 100,000 in 2023, and the overall prevalence increasing from approximately 396 to 523 per 100,000 during the same time period (6). According to the Centers for Disease Control and Prevention (CDC), IBD affects an estimated 2.4–3.1 million individuals in the United States (U.S.), with variations in burden across demographic groups. In 2018, total annual healthcare expenses for IBD in the U.S. were approximately $8.5 billion, underscoring its substantial health and economic effects (7). The incidence of IBD has steadily increased in the Western world during the twentieth century. Although it has leveled off in certain areas, it continues to rise among specific groups, including pediatric-onset cases. Currently, IBD affects approximately 0.5% of the population in Western nations, with more than 1 million cases in the U.S. and 2.5–3.0 million in Europe. Although previously rare in developing regions, IBD has rapidly emerged in newly industrialized nations in Asia, South America, and the Middle East, where its incidence is increasing significantly. Due to its persistent nature and low mortality, IBD exhibits “compounding prevalence,” resulting in a continuous and exponential increase in the worldwide disease burden (8).

Treatment for IBD has traditionally relied on corticosteroids, aminosalicylates, and immunosuppressive medications. Corticosteroids such as hydrocortisone, prednisolone, and budesonide are used to induce remission by decreasing inflammation via the inhibition of the NF-κB pathway; however, prolonged use is associated with various side effects, such as weight gain, swelling, acne, increased appetite, insomnia, dyspepsia, and mood changes (9). Aminosalicylate derivatives, such as Olsalazine, Balsalazide, and Sulfasalazine, are frequently recommended for mild to moderate UC but are less effective in CD. These agents act by blocking NF-κB, reducing oxidative stress, and altering arachidonic acid metabolism, thereby reducing the production of inflammatory substances such as prostaglandins and leukotrienes. Consequently, 5-ASA reduces intestinal inflammation and protects epithelial cells. Nonetheless, 5-ASA treatment has been associated with negative effects, such as pancreatitis, heart toxicity, liver and kidney damage, sexual issues, gastrointestinal problems, and skin reactions (10). Immunosuppressants, particularly anti-TNF medications such as Infliximab, Adalimumab, Certolizumab pegol, and Golimumab have improved IBD management by providing more precise anti-inflammatory effects. These agents reduce inflammation by inhibiting tumor necrosis factor (TNF) signaling and restricting immune cell infiltration (11). However, some patients may not initially respond to treatment, others may lose their response over time, and repeated therapies may result in immunogenicity, highlighting the importance of patient selection (12, 13). Current IBD treatments encompass anti-integrin agents (like vedolizumab, natalizumab) which block leukocyte migration, IL-12/23 and IL-23 inhibitors (focusing on p40 or p19 units) that reduce inflammatory cytokine signaling, JAK inhibitors (such as tofacitinib, upadacitinib) that disrupt intracellular cytokine pathways, and S1P receptor modulators (for example, ozanimod, etrasimod) that control lymphocyte movement (14–16). Despite advancements in these therapies for IBD, issues like insufficient response, side effects, high expenses, and long-term safety worries underscore the necessity for additional and alternative treatment options. Hence, there is an urgency for alternative therapeutic approaches with greater effectiveness and minimal side effects to complement the existing IBD treatments.

Ginseng could be a potential therapeutic agent for IBD owing to its strong anti-inflammatory and immunomodulatory effects. *In vitro*, and *in vivo* studies have revealed that ginseng can reduce intestinal inflammation by influencing immune cell differentiation, inhibiting inflammatory cytokine production, and modulating key inflammatory signaling pathways (17–20). Ginseng also play a role in maintaining immune balance, strengthening the intestinal barrier, and rehabilitating the gut microbiota (21–23). Ginseng and its bioactive compounds exhibit diverse biological and pharmacological activities, including anti-inflammatory, immunomodulatory, and antioxidant effects (24, 25). Moreover, traditionally valued as a medicinal herb, ginseng has been widely used and scientifically investigated for the treatment of various conditions, including diabetes mellitus, fatigue, tumors, ulcers, hypertension, stress, and neurological diseases, such as Alzheimer’s, Parkinson’s, and Huntington’s diseases (26–32). Ginseng contains numerous bioactive compounds, including ginsenosides, polysaccharides, peptides, fatty acids, polyacetylenes, and gintonin. Ginsenosides are considered the most significant and active of these components, with over 100 varieties recognized to date. These substances demonstrate notable anti-inflammatory effects and have displayed beneficial effects in a range of inflammatory conditions (33–35). Therefore, ginsenosides, considered the main bioactive compounds, are thought to contribute to its health benefits and pharmacological effects of ginseng. However, despite their encouraging preclinical results, their clinical application is impeded by low oral bioavailability, inadequate intestinal uptake, and reliance on metabolism mediated by complex gut microbiota. In addition, variability in pharmacokinetics across individuals and species, differences in structure, and variability between products lead to inconsistent therapeutic results. The scarcity of well-designed clinical evidences also restricts the validation of their effectiveness in human IBD (36–39). Therefore, enhancing the bioavailability of ginsenosides is an important and utmost attention for future IBD research. In this review, we aim to explore the role of ginseng and its derivatives in IBD management and provide a theoretical basis for the development of prospective therapeutic strategies and potential approaches to overcome existing limitations. Most of the evidence covered in this review derived from UC models; hence, the impacts of ginseng and its bioactive components should be interpreted mainly within the framework of UC.

## 2 Methods

To find pertinent research on the therapeutic potential of ginseng and its bioactive constituents in inflammatory bowel disease (IBD), a thorough literature search was carried out. Combinations of keywords like “ginseng,” “Panax species,” “ginsenosides,” “ginseng polysaccharides,” “bioactive compounds,” “IBD,” “Crohn’s disease,” “ulcerative colitis,” “colitis,” “inflammation,” “gut microbiota,” “ginsenosides and ginseng alleviate IBD,” and “intestinal barrier” were used to retrieve published articles from databases such as PubMed, Scopus, Web of Science, and Google Scholar. Studies that focused on IBD pathogenesis, structure and characteristics of ginseng, the mechanisms of action, anti-inflammatory properties, immune regulation, gut microbiota modulation, and intestinal protective benefits of substances derived from ginseng were chosen. An overview of the present knowledge and therapeutic potential of ginseng in IBD was produced by analyzing and summarizing the collected evidences.

## 3 Chemistry and classification of ginsenosides

**Figure 1:**
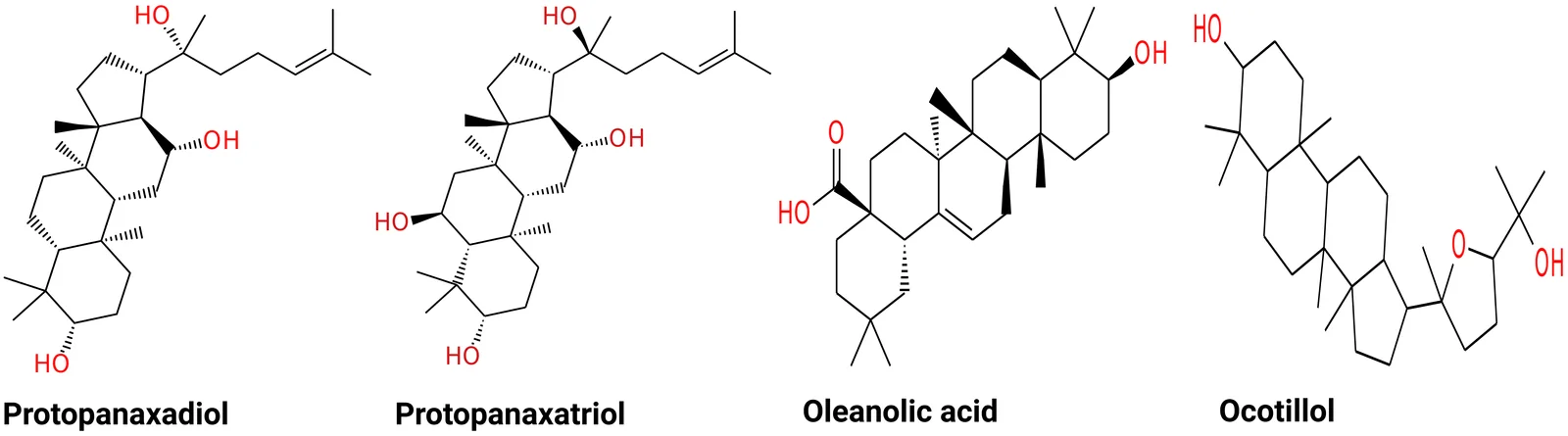
Schematic representation of the chemical structure of different ginsenosides.

Ginsenosides are distributed throughout the ginseng plant, including in the roots, leaves, stems, flowers, and fruits. Their composition and content vary according to the ginseng species and plant parts (40). Ginsenosides are saponins, which are glycosylated triterpenoids featuring a hydrophobic aglycone framework with approximately 30 carbon atoms and one or more hydrophilic sugar units, including glucose, rhamnose, xylose, and arabinose. These sugar moieties are often linked to specific carbon sites (primarily C-3, C-6, and C-20), which enhances their structural diversity and biological roles (41–43). Ginsenosides are mainly categorized into dammarane- and oleanane-type saponins based on their chemical structures and aglycone frameworks (**Table 1**). Dammarane-type ginsenosides comprise the bulk and are categorized into protopanaxadiol (PPD) and protopanaxatriol (PPT) groups, based on the location and quantity of hydroxyl groups and connected sugars. Other minor types include ocotillol-type ginsenosides with distinct epoxy ring structures and oleanane-type ginsenosides with pentacyclic structures, which are found in smaller quantities (44–46). The structural differences among these categories, particularly the changes in the aglycone backbone and sugar chains, are crucial for influencing the pharmacological effects and therapeutic potential of ginsenosides (47) (**Figure 1**).

**Table 1:** Classification of ginsenosides.

Category	Backbone	Examples
Protopanaxadiol	Dammarane	Rb1, Rb2, Rb3, Rc, Rd, Rg3, Rh2, Rs
Protopanaxatriol	Dammarane	Re, Rf, Rg1, Rg2, Rh
Oleanolic acid	Pentacyclic triterpenoid	Ro
Ocotillol	Five-membered epoxy ring at C20	F11, RT2, RT4, RT5

## 4 Mechanistic basis of IBD pathogenesis

**Figure 2:**
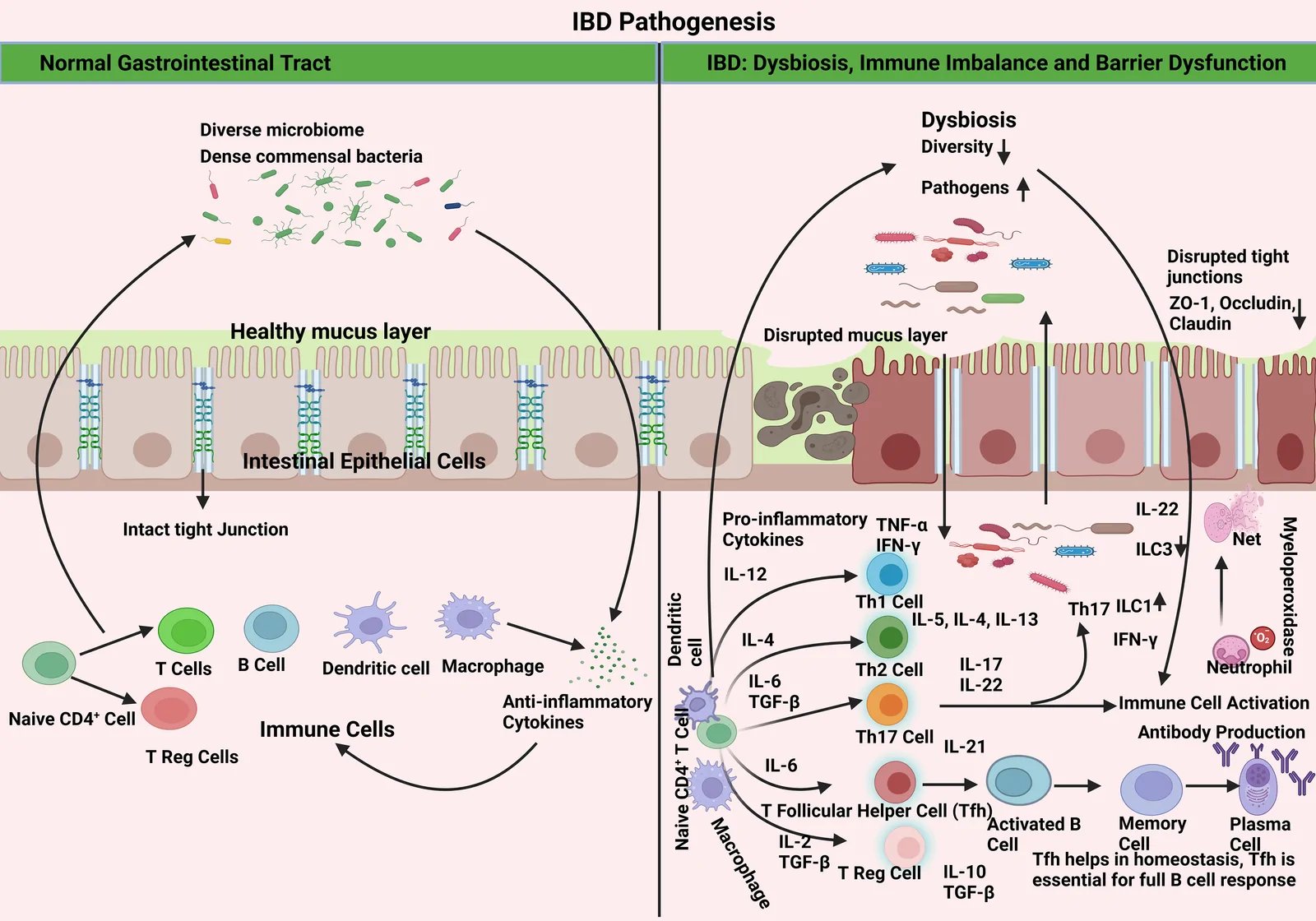
Mechanistic insights into the pathogenesis of inflammatory bowel disease (IBD). The development of IBD is driven by complex interactions among intestinal barrier disruption, dysfunctional immune cell differentiation, dysregulated cytokine production and signaling pathways, and gut microbiota alterations. These factors collectively lead to sustained immune activation and chronic intestinal inflammation. (↓: Decrease; ↑: Increase) (Created with Biorender.com).

The intestinal mucosa maintains stability with luminal contents through the intestinal barrier, composed of epithelial, microbial, chemical, and immune components. This selective barrier facilitates nutrient uptake while regulating the entry of pathogens, toxins, and antigens, thus preserving immune balance. Disruption of barrier integrity increases intestinal permeability, allowing harmful agents to penetrate the mucosa and trigger inflammatory reactions (48–51). Although genetic predisposition and environmental factors may contribute to disease development, the disruption of the epithelial barrier and subsequent immune activation are considered central events in the onset and progression of IBD (52). Its pathogenesis involves intricate interactions between intestinal endothelial and immune cells, abnormal cytokine production and signaling pathways, and disruption of the gut microbiome, resulting in persistent immune activation and chronic intestinal inflammation (**Figure 2**).

### 4.1 Intestinal epithelial cells (IECs)

The intestinal epithelium consists of a single cell layer that lines the intestinal tract, creates a physical barrier, and is crucial for preserving barrier integrity and immune balance (53). The specialized IECs, such as absorptive, goblet, enteroendocrine, Paneth, M, cup, and tuft cells, play roles in mucus secretion, antimicrobial defense, immune regulation, and interaction with the underlying immune system (54). The integrity of the epithelial barrier is maintained by tight junctions, mucus layers, and antimicrobial peptides that regulate intestinal permeability and block microbial invasion (55). In IBD, breakdown of epithelial integrity, including altered tight junctions, increased apoptosis of IECs, and dysfunctional differentiation of epithelial cell types, results in enhanced intestinal permeability and irregular immune activation (56, 57). Pro-inflammatory cytokines, such as tumor necrosis factor-α (TNF-α), interleukin (IL)-1β, IL-6, and interferon-γ (IFN-γ), also enhance epithelial apoptosis and compromise barrier function (58–60). Consequently, epithelial damage facilitates the entry of microbes into the mucosa, triggering the immune response and leading to ongoing inflammation, establishing intestinal epithelial dysfunction as a key factor in IBD development (61, 62).

### 4.2 Intestinal immune cells

Dysregulated immune responses in the digestive system are a hallmark of IBD. Innate immunity provides the first-line defense through pattern recognition receptors (PRRs) and consists of IECs, Paneth cells, macrophages, (Dendritic cells) DCs, neutrophils, monocytes, (Innate lymphoid cells) ILCs, and (Natural killer cells) NK cells, which block pathogens while maintaining tolerance for commensal microbiota (63). Nonetheless, alterations in this immune equilibrium cause heightened inflammatory responses that contribute to chronic intestinal inflammation and develop IBD (64). In UC, activated neutrophils accumulate in the intestinal mucosa and promote inflammation by secreting neutrophil extracellular traps and reactive oxygen species via enzymes, such as myeloperoxidase. This results in epithelial damage, crypt abscess formation, and ulceration (65, 66). In IBD, NK cells show metabolic impairment with diminished mitochondrial function and decreased mTORC1 activity, even though pro-inflammatory IL-17A and TNF-α production is elevated. They additionally control neutrophil-driven inflammation through the inhibitory receptor NKG2A (67, 68). ILCs are crucial for intestinal immunity, barrier maintenance, and tissue repair. Nonetheless, the imbalance among ILC subsets contributes to disease development. Elevated ILC1 levels lead to inflammation via cytokines such as IFN-γ, reduced ILC3 levels weaken the intestinal barrier protection usually provided by IL-22, and altered ILC2 activity may either worsen inflammation or aid epithelial repair (69). In IBD, DCs transition from tolerogenic to pro-inflammatory states. Typically, intestinal CD103^+^ DCs sustain immune tolerance by producing IL-10 and facilitating the differentiation of Foxp3^+^ regulatory T cells (Tregs) (70). In IBD, the number of pro-inflammatory DCs increases, whereas that of the tolerogenic subsets decreases. These DCs exhibit increased expression of toll-like receptor 2 (TLR2) and TLR4, triggering NF-κB signaling and generating cytokines, such as IL-12, IL-23, and TNF, which enhance Th1 and Th17 responses and contribute to chronic intestinal inflammation (71, 72). In healthy subjects, intestinal macrophages promote immune tolerance by exhibiting robust phagocytic activity and releasing anti-inflammatory cytokines such as IL-10, which bolster Treg responses and maintain gut homeostasis (73). In IBD, this equilibrium is disrupted, leading to the accumulation of pro-inflammatory macrophages, particularly M1 macrophages. These cells produce elevated levels of inflammatory mediators, including TNF-α, IL-6, IL-1β, and IL-23, which enhance Th1 and Th17 immune responses and intensify intestinal inflammation (74, 75). Additionally, increased levels of CD14^+^ and TREM-1^+^ macrophages boost cytokine release and inflammatory signaling, leading to chronic mucosal inflammation and tissue injury (76, 77).

Adaptive immune cells, such as T helper cells (Th1, Th2, Th17, and Th9) and B cells, play an important role in the pathogenesis of IBD. Unlike innate immunity, adaptive immunity is specific, long-lasting, and capable of immune memory. However, it develops slowly and with greater precision. CD8^+^ T cells contribute to IBD pathogenesis through distinct functional subsets. Cytotoxic CD8^+^ T cells (Tc1) and IL-17–producing CD8^+^ T cells (Tc17) promote inflammation by secreting IFN-γ and TNF-α, resulting in IEC injury, whereas tissue-resident memory CD8^+^ T cells (Trm) may exert context-dependent regulatory effects, and exhausted CD8^+^ T cells have been correlated with improved clinical outcomes (78, 79). Similarly, naïve CD4^+^ T cells differentiate into distinct Th-cell subsets in response to cytokine milieu and transcription factor activation. Upon exposure to antigens, they can differentiate into effector T-cell subsets, such as Th1, Th2, Th9, Th17, Th22, T follicular helper (Tfh) cells, or Tregs (80). In CD, Th1 cells release elevated levels of TNF-α and IFN-γ, thus activating macrophages and exacerbating intestinal inflammation (81). In contrast to UC, tissues in CD demonstrate increased IFN-γ and IL-12 expression, suggesting a more robust Th1-mediated immune response (82). In UC, the immune response is often described as a Th2-driven phenomenon. Patients exhibit elevated IL-5 and IL-13 secretions, whereas IL-4 levels are comparatively lower (83). Th2 cell activation occurs via STAT6 signaling induced by IL-4 and the transcription factor GATA-3, with additional enhancement by IL-33 in intestinal tissues (84, 85). IL-13, primarily produced by NK-T and Th2 cells, compromises the intestinal epithelial barrier by modulating tight junctions, promoting epithelial apoptosis, and hindering mucosal repair, thereby facilitating inflammation. Excessive Th2 cell activation may lead to persistent inflammation and tissue damage in UC (86). Th9 cells play a significant role in the development of UC. They differentiate from naïve T cells in the presence of IL-4 and TGF-β, primarily generating IL-9. IL-9 enhances mucus secretion, promotes neutrophil migration, and increases epithelial permeability by affecting tight junction proteins (87). A study reported that, TH9-derived IL-9 induces chronic intestinal inflammation and contributes to the pathophysiology of UC by impairing intestinal barrier function and inhibiting mucosal wound healing. Its neutralization lessens disease severity, weight loss, and histological injury, suggesting IL-9 as a possible therapeutic target (88). CD4^+^ T cells differentiate to Th17 cells via the STAT3 signaling pathway, particularly when cytokines such as IL-6, TGF-β, IL-21, and IL-23 are present (89). In IBD, elevated levels of IL-23 and IL-17 promote the growth of Th17 cells. These cells produce inflammatory cytokines, including IL-17A, IL-17F, IL-21, IL-22, IL-12, and TNF-α, which enhance inflammation (90). Th22 cells produce IL-22, which helps maintain the intestinal epithelial barrier, promotes epithelial cell proliferation, and supports tissue repair and antimicrobial defense (91, 92). In UC, the proportion of Th22 cells decreases, whereas that of Th17 cells increases, indicating a disrupted Th17/Th22 balance that contributes to intestinal inflammation (93). Tregs maintain intestinal immune tolerance and regulate inflammation during IBD. Unlike effector T cells, Tregs differentiate in response to IL-2 and TGF-β, leading to Foxp3 expression and increased production of anti-inflammatory cytokines, such as IL-10, IL-35, and TGF-β. These cytokines inhibit inflammatory pathways and control the equilibrium between Treg and Th17 cells (94, 95). In IBD, while Treg populations can increase in intestinal tissues, their suppressive capacity is frequently compromised by faulty signaling, diminished migration, or increased apoptosis (96). In IBD, increased Tfh cell numbers correlate with disease severity. They promote intestinal inflammation by increasing B-cell activation, antibody production, and cytokine secretion (IL-4 and IL-21) and may also trigger colitis via B-cell–independent pathways, including BCL-6. In contrast, T follicular regulatory (Tfr) cells, which usually inhibit Tfh responses, are diminished, resulting in an imbalance that promotes disease progression and can serve as a biomarker of disease activity (97). In addition to T cells, B cells significantly contribute to IBD development. Typically, B cells maintain intestinal homeostasis by producing IgA, which regulates the gut microbiota and safeguards the epithelial barrier (98). In IBD, dysregulated B-cell responses are characterized by elevated antibody production (notably IgG), abnormal responses to bacterial antigens, and heightened inflammatory signaling (99). In UC, there is a decrease in regulatory B cells (Bregs) that produce IL-10 and an increase in inflammatory plasma cells that secrete IgG, leading to enhanced recruitment of immune cells and intestinal inflammation (100).

### 4.3 Gut microbiota

Gut microbiota dysbiosis is crucially associated with IBD and is characterized by a decline in microbial diversity and an imbalance between beneficial and pathogenic microorganisms. The gut microbiota of healthy individuals is primarily abundant in beneficial bacteria, particularly Firmicutes and Bacteroidetes, which sustain intestinal homeostasis by generating SCFAs such as butyrate, modulating immune responses, and reinforcing epithelial barrier integrity (101). In contrast, individuals with IBD typically show a reduced abundance of beneficial SCFA-producing bacteria, such as *Faecalibacterium prausnitzii, Roseburia, Eubacterium rectale*, and *Bifidobacterium longum*, while also displaying elevated levels of potentially harmful bacteria, particularly Proteobacteria and *Escherichia coli* (notably adhesion-invasive *E. coli*, AIEC) (102–104). This microbial perturbation depletes SCFA production and tryptophan metabolites, restricts Treg differentiation, compromises epithelial barrier integrity, innate immune cell activation, and facilitates inflammatory cytokine production (104, 105).

Additionally, elevated levels of mucolytic bacteria (e.g., *Ruminococcus gnavus and Ruminococcus torques*) and sulfate-reducing bacteria (e.g., *Desulfovibrio*) can harm the intestinal mucosa by degrading mucus and producing toxic metabolites such as hydrogen sulfide (106, 107). In addition to bacteria, dysbiosis involves changes in fungal, viral, and archaeal communities. These alterations in microbiota hinder immune regulation, increase intestinal permeability, and initiate chronic inflammation through interactions with host immune pathways (108). In summary, dysbiosis of the gut microbiota affects IBD development by altering the microbial ecosystem, metabolic activities, and interactions between the host and microbes, ultimately resulting in epithelial barrier dysfunction and intestinal inflammation.

## 5 Therapeutic role of ginseng and its bioactive compounds in the management of IBD

**Figure 3:**
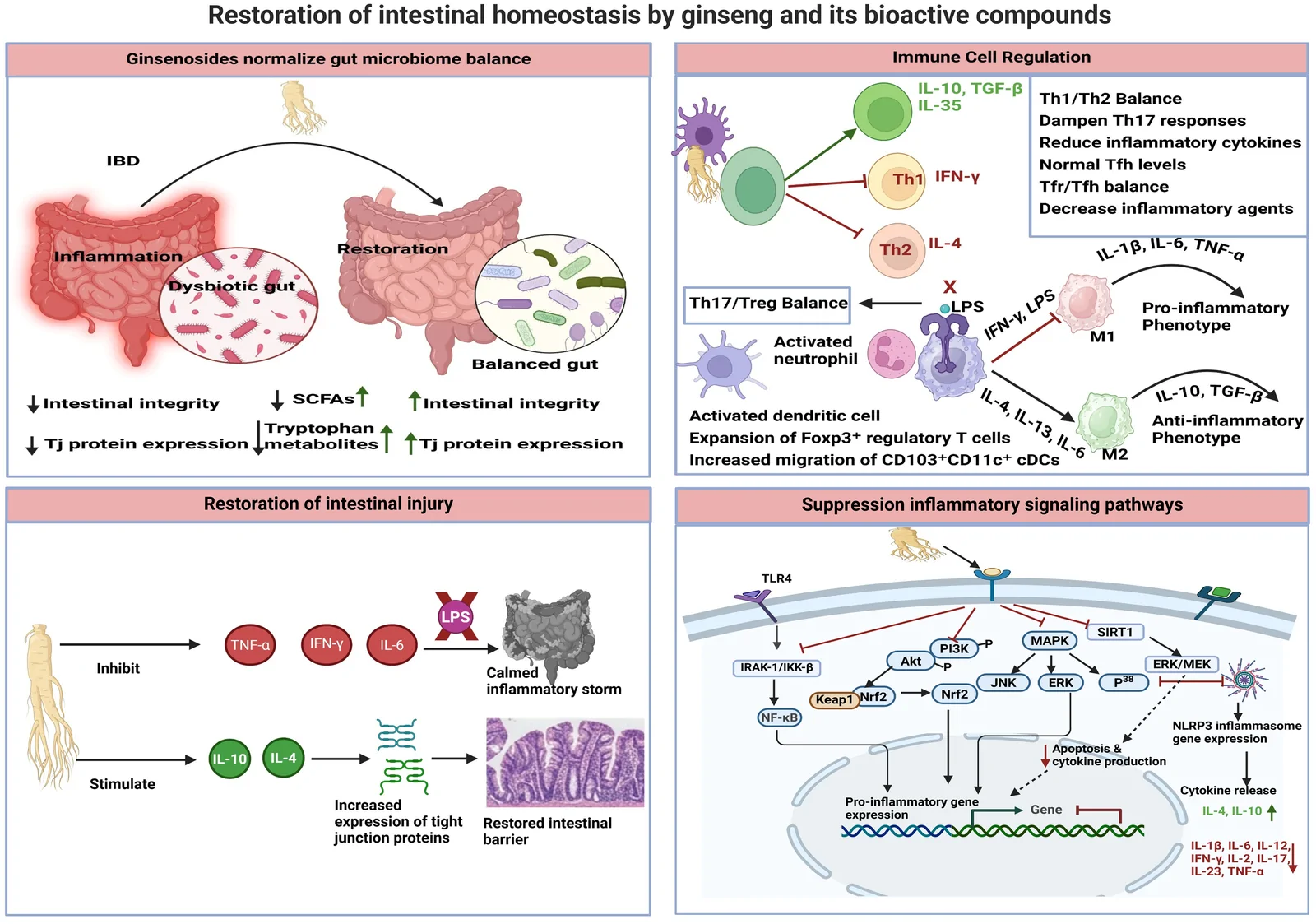
Ginseng and its bioactive compounds restore intestinal homeostasis disrupted by IBD. The therapeutic effects of ginsenosides are mediated by diverse mechanisms, including immune cell modulation, cytokine regulation, repair of intestinal injury, suppression of inflammatory signaling pathways, and normalization of gut microbial balance. (↓: Decrease; ↑: Increase; ⊣: Inhibition; X: Blocking). (Created with Biorender.com).

*In vitro* and *in vivo* studies demonstrated that ginseng and its derivatives possess pharmacological activity and has therapeutic potential in IBD management. Accumulating evidences indicate that ginsenosides ameliorate experimental colitis through diverse mechanisms, including immune cell modulation, cytokine regulation, repair of intestinal pathological injury, suppression of inflammatory signaling pathways, and normalization of the gut microbial balance (**Table 2**; **Figure 3**).

**Table 2:** Overview of preclinical evidence on ginseng-derived compounds in experimental IBD models, emphasizing their protective roles through various mechanisms.

Ginsenoside/ginseng derivatives	Type	IBD model	Dose	Route	Duration	Result	Mechanism	Evidence level
Red ginseng ethanolic extract (R1, Rb1, Rb2, Rh1, Rh4, and Rh2)	PPD and PPT	DSS-induced colitis mouse	100 mg/kg	Oral administration	10 days	Reduced colitis severity, histological damage, and inflammatory cytokines	Anti-inflammatory functions via suppression of NO, TNF-α, IL-6, and IL-1β; possible contribution of ginsenosides	Preclinical (109)
Ginseng polysaccharides	Polysaccharide (non-saponin)	DSS-induced colitis mouse	50, 100, 200 mg/kg/day (optimized dose: 200 mg/kg)	Oral administration	10 Days	Enhanced colonic pathology, decreased inflammatory cytokines and inflammatory cell infiltration, strengthened intestinal barrier integrity.	Adjusted gut microbiota, modified microbial tryptophan metabolites, and blocked 5-HT/HTR3A signaling to regain barrier function.	Preclinical (110)
Ginseng polysaccharides	Polysaccharide (non-saponin)	DSS-induced IBD model in dogs	350 mg/kg	Oral administration	20 Days	Enhanced clinical symptoms, colonic histopathology, and lowered inflammatory marker CRP; normalized gut microbiota balance.	Altered gut microbiota (↑*Bacteroides, Megamonas, Fusobacterium; ↓Campylobacter*) and enhanced microbial metabolic activities	Preclinical (111)
Ginseng polysaccharides	Polysaccharide (non-saponin)	DSS-induced colitis mouse model	50 and 100 mg/kg/day	Oral administration	7 days	Decreased disease activity index, shortened colon, histological injury, oxidative stress, and inflammatory cytokines.	Inhibit JAK2/STAT1/NLRP3 inflammasome signaling pathway to reduce inflammation	Preclinical (112)
Rg1	PPT	Obesity-associated colitis mouse model	200 mg/kg/day	Oral administration	21 Days	Reduced colitis severity, enhanced lipid metabolism and liver function.	Regulate gut microbiome, T-cell responses and improve lipid metabolism	Preclinical (113)
Rg1	PPT	DSS-induced colitis mouse	200 mg/kg	Oral administration	9 Days	Increased body mass, reduced colonic injury scores and disease activity index, along with decreased proinflammatory cytokine levels	Regulate NLRP12 expression.	Preclinical (114)
Rb1	PPD	TNBS-induced colitis in male Wistar rats	Not indicated for isolated Rb1 (Daikenchuto: 900 mg/kg/day)	Oral administration	7 Days	Improved colonic epithelial restoration and promoted mucosal healing	Stimulated intestinal epithelial wound repair through ERK and Rho signaling pathways	Preclinical (115)
Rb1	PPD	DSS-induced and TNBS-induced colitis mouse models (TNBS model has Crohn’s-like features)	20 and 40 mg/kg/day	Oral administration	7 Days	Decreased colitis severity, tissue damage, inflammation, and epithelial cell death.	Stimulated Hrd1 signaling, decreased ER stress indicators (GRP78, PERK, CHOP, caspase-12), and blocked Fas-induced apoptosis.	Preclinical (116)
RT4	Ocotillol	DSS-induced colitis mouse	5, 10, 20 mg/kg			Decreased weight, shortening of colon length, damage to the colonic bowel, and index of disease activity	the miR-144-3p/SLC7A11 signaling pathway to reduce inflammation and ease colitis.	Preclinical (117)
Rd	PPD	Indomethacin-induced IBD rat	10, 20, or 40 mg/kg per day (optimal results at 20 mg/kg)	Oral administration	7 Days	Decreased intestinal injury and improved mucosal restoration	Stimulated the growth and maturation of native intestinal stem cells to regain intestinal function.	Preclinical (118)
Compound K	PPD	AOM/DSS-induced colitis-associated colorectal cancer (CAC) in Balb/c mice	30 and 60 mg/kg (optimized at 60 mg/kg)	Oral administration (mixed with chow)	45 Dyas	Inhibited tumor progression and enhanced gut microbiota ecosystem	Increased abundance of *Akkermansia muciniphila*	Preclinical (119)
Rk3	PPT	Antibiotic-induced gut microbiota dysbiosis and intestinal inflammation mouse model	20 and 60 mg/kg	Oral administration	2 Weeks	Improved intestinal inflammation, reduced inflammatory cytokines, and restored gut barrier integrity	Altered gut microbiota, elevated SCFA levels, and increased expression of tight junction proteins	Preclinical (120)
Rk3	PPT	High-fat diet-induced colitis C57BL/6 mice	60 mg/kg	Oral administration	8 Weeks	Reduced colonic inflammation, oxidative stress, and lipid metabolic issues	Controlled polyunsaturated fatty acid metabolism; suppressed COX/LOX pathways, diminished pro-inflammatory lipid mediators (PGE2, PGD2, TXB2, HETE, HODE), and enhanced anti-inflammatory metabolites (EET, diHOME)	Preclinical (121)
Ginsenoside F2	PPD	DSS-induced colitis mouse model	50 and 100 mg/kg/day	Oral administration	Not specified	Reduced colitis symptoms, restore gut barrier function, and reduced inflammation	Suppressed NLRP3 inflammasome activation by inhibiting NLRP3 assembly and associated protein expression.	Preclinical (122)
Rf	PPT	TNF-α-stimulated intestinal epithelial cells (HT-29) and mouse macrophage cells (RAW264.7)	Not specified (up to 100 μM without cytotoxicity)	Cell treatment	Not specified	Reduced inflammatory mediators	Inhibit p38/NF-κB signaling and NF-κB transcriptional activity	Preclinical (123)
Rh2	PPD	DSS-induced colitis mouse model	20 mg/kg	Oral administration	7 Days	Reduce colitis symptoms and colon damage	Increased TGF-β signaling, which facilitated the protective effect against colitis.	Preclinical (124)

### 5.1 Immune modulation

IBD is a multifactorial, immune-mediated chronic disorder in which genetic, environmental, and immune factors drive excessive intestinal inflammation mediated by T cells. Ginseng and its bioactive compounds, particularly ginsenosides ameliorate experimental colitis symptoms by modulating immune cell functions. They help normalize immune balance by suppressing pro-inflammatory T-cell subsets such as Th1 and Th17, while promoting Treg proliferation (125). Ginsenoside Rg3 regulates the Th1/Th2 cytokine balance and exerts strong anti-inflammatory effects, thereby reducing the severity of UC. It interferes with the MAPK and NF-κB signaling pathways, reduces pro-inflammatory cytokine production, suppresses NLRP3 inflammasome activation, and facilitates colon length and histopathological damage in dextran sodium sulfate (DSS)-induced colitis models (126). In UC, immune dysregulation is marked by elevated number of Tfh cells, decreased number of Tregs, and an altered Tfr/Tfh ratio. Ginsenoside Rb1 mitigates DSS induced experimental colitis by reestablishing the Tfh/Treg equilibrium, likely through the suppression of the PI3K/AKT pathway. It diminishes pro-inflammatory Tfh9 and Tfh17 cell levels while elevating Tfr and Treg populations (127). Ginsenoside Rg1 and its metabolites, Rh1 and 20(S)-protopanaxtriol, inhibit the interaction of lipopolysaccharide (LPS) with TLR4 on macrophages, restore the Th17/Treg balance, and reduce the abundance of inflammatory cytokines (IL-1β, IL-17, and TNF-α), thus ameliorating experimental colitis induced by 2,4,6-trinitrobenzene sulfonic acid (TNBS) (128). Additionally, ocotillol ameliorates TNBS colitis by normalizing the Th17/Treg balance, dampening Th17 responses, boosting Treg function, inhibiting inflammatory cytokine and signaling pathways, decreasing inflammatory mediators, and enhancing intestinal barrier integrity (129). Moreover, ginseng berry (GB) extract reduces DSS-induced colitis by inhibiting the infiltration and activation of inflammatory immune cells (T cells, neutrophils, DCs, and macrophages) and promoting immune regulation via increased migration of CD103^+^CD11c^+^ cDCs and expansion of Foxp3^+^ Tregs. It also enhances colon morphology, indicated by the increased colon length, and decreased histological damage (130). STAT3 plays a crucial role in T-cell differentiation and promotes the expression of pro-inflammatory cytokines. *P. notoginseng* mitigates DSS-induced colitis by inhibiting STAT3, reducing pro-inflammatory cytokine production, and restoring the Th17/Treg balance (131). Similarly, the Ginsenoside Rh2 demonstrates potent anti-inflammatory properties by suppressing the STAT3/miR-214 signaling pathway. It relieves symptoms of DSS-induced colitis, such as weight loss, intestinal injury, colon shortening, and disease activity index (DAI) scores, while lowering the abundance of pro-inflammatory cytokines (TNF-α, IL-6, and IL-1β). *In vitro* studies have demonstrated the inhibition of IL-6–induced STAT3 activation and miR-214 expression in colonic epithelial cells (132). Macrophages polarize into pro-inflammatory M1 and anti-inflammatory M2 phenotypes, and excessive M1 activation contributes to colitis. Ginsenoside Rg1 reduces the overall and activated macrophage populations (CD11b^+^F4/80^+^, CD11b^+^F4/80^+^Tim-1^+^, and CD11b^+^F4/80^+^TLR4^+^) alongside pro-inflammatory M1 macrophages (CD11b^+^F4/80^+^iNOS^+^), while enhancing anti-inflammatory M2 macrophages (CD11b^+^F4/80^+^CD206^+^ and CD11b^+^F4/80^+^CD163^+^) in DSS induced colitis BALB/c mice, thus reestablish the M1/M2 balance (133). Additionally, fermented red ginseng (FRG) exerts potent immunomodulatory and anti-inflammatory effects in TNBS induced colitis mice by suppressing macrophage activity and regulating the Th1/Treg balance (134). These findings suggest that ginsenosides have therapeutic potential for managing IBD by modulating immune cell proliferation and differentiation and regulating cytokine production.

### 5.2 Barrier repair

Disruption of the intestinal barrier is a hallmark of IBD. Therefore, maintaining and restoring barrier stability is essential for IBD management. Ginsenosides repair the disrupted epithelial barrier by normalizing barrier integrity and reducing inflammation (135). *In vivo* studies in DSS-induced colitis animal models suggest that various ginseng derivatives may improve colitis-associated symptoms through the protection or restoration of intestinal barrier integrity. Ginsenoside Rb1 improves intestinal barrier integrity by elevating the expression of tight junction proteins, such as Zonula Occludens-1 (ZO-1), Occludin, and E-cadherin, which are essential for sustaining intestinal barrier integrity, while decreasing the levels of pro-inflammatory cytokines (TNF-α and IL-6) and increasing the level of anti-inflammatory cytokine IL-10, thus ameliorating colitis symptoms (136, 137). Ginsenoside Rg1 restores the integrity of the intestinal epithelium by decreasing the levels of pro-inflammatory cytokines (TNF-α and IFN-γ) and elevating the level of IL-4, thereby ameliorating colitis (138). Moreover, it protects tight junctions against LPS-induced tight junction disruption by enhancing ZO-1, Occludin, and Claudin-1 levels and suppressing the p38 MAPK-mediated NLRP3 inflammasome pathway in a porcine model (139). Similarly, other ginsenosides, including Rk2, Rk3, and Rc improve gut barrier function by upregulating the expression of tight junction proteins in DSS induced experimental colitis models (140–142).

### 5.3 Signaling pathways

Inflammatory signaling pathways in IBD are linked to each other instead of being separate. MAPK (ERK/JNK/p38) and PI3K/Akt serve as key upstream signaling pathways, whereas IRAK-1 and IKK-β facilitate receptor-induced activation of NF-κB. NF-κB and STAT control the expression of inflammatory genes, while NLRP3 acts as a downstream effector of the inflammasome, and Nrf2/HO-1 alongside TGF-β/Smad mainly facilitate antioxidant responses and tissue repair (143, 144). The dysregulated activation of these signaling pathways promotes inflammation and facilitates the onset and progression of IBD. Ginsenosides improve IBD by positively regulating these inflammatory pathways. Ginsenosides Rf in DSS induced colitis and Re in LPS-treated RAW264.7 cells exert anti-inflammatory effects by suppressing NF-κB-dependent signaling pathways. Treatment with DSS and LPS upregulates NF-κB expression and induces elevated levels of iNOS, COX-2, TNF-α, and IL-6, whereas Rf and Re inhibit NF-κB expression, resulting in a notable reduction in these inflammatory mediator levels (145, 146). Additionally, fermented wild ginseng suppresses inflammatory responses by inhibiting NF-κB transcriptional activity, specifically by reducing nuclear translocation in DSS-induced mice and LPS-stimulated RAW264.7 macrophages. This indicates that its anti-colitic effects are likely linked to the inhibition of NF-κB–mediated macrophage activation (147). Ginsenosides suppress NF-κB activation in a Pregnane X receptor (PXR)-dependent manner by decreasing p65 nuclear translocation and normalizing PXR/NF-κB interaction. They also restore disrupted PXR/NF-κB signaling during the inflammatory state, thereby ameliorating colitis in mice induced by DSS (148). Ginsenoside Rb1 and its metabolite Compound (CK) reduced pro-inflammatory cytokine production, suppressed the IRAK-1/NF-κB/MAPK signaling pathway, and improved the severity of colitis in TNBS-induced colitis and LPS-stimulated murine macrophages (149). Similarly, in TNBS-induced colitis mice and LPS-stimulated macrophages, ginsenoside Re inhibits the TLR4/NF-κB pathway by disrupting the LPS–TLR4 interaction, thereby preventing the activation of IRAK-1/IKK-β (150). In a mouse model of UC induced by oxazolone, the combination of Korean Red Ginseng extract enriched with Rg3 and *Persicaria tinctoria* demonstrated synergistic anti-inflammatory effects by inhibiting NF-κB and NLRP3 signaling while reestablishing immune cell balance, including CD4^+^ T cells, CD19^+^ B cells, and regulatory T cells (Tregs) (151). Additionally, *P. notoginseng* saponins (PNS) reduce inflammation by blocking the PI3K/AKT signaling pathway in DSS induced colitis rats (152). Moreover, ginsenoside Rd diminishes inflammation in TNBS induced colitis rat model by modulating the pathways associated with oxidative stress and inflammatory mediators (153).

A crucial process involves suppressing the NLRP3 inflammasome, which is vital for intestinal inflammation. *In vivo*, ginsenosides Rg1, Rg2, Rb1, and Rd inhibit NLRP3 activation through various upstream regulators, thereby reducing caspase-1 activation, IL-1β synthesis, and pyroptosis (137, 139, 154). Various ginsenosides modulate MAPK signaling pathways to regulate inflammation. In LPS-induced epithelial injury and TNBS-induced colitis models, Rg1 and Rd block p38 MAPK, with Rd additionally reducing JNK signaling (139, 155), whereas in antibiotic-induced gut dysbiosis models Rh4 affects the TLR4–MyD88–MAPK pathway, collectively decreasing pro-inflammatory cytokine production and leukocyte influx (156). In a Caco-2/THP-1 co-culture model of experimental colitis ginsenoside Rk2 reduces inflammation by blocking the SIRT1-driven ERK/MEK pathway, thereby lowering apoptosis and cytokine production (157). In addition to their direct anti-inflammatory properties, ginsenosides regulate oxidative stress and cellular quality control processes. In DSS colitis mice Rb1 stimulates the Nrf2/HO-1 pathway, boosting antioxidant defenses and reducing inflammasome activation, whereas Rd promotes AMPK/ULK1-related autophagy (mitophagy) to block NLRP3 signaling (137, 158). Additionally, Rb1 influences the PIP2/Ca²^+^ pathway, thereby diminishing pyroptosis (137). Significantly, these signaling pathways lead to functional improvements, such as the recovery of intestinal barrier integrity (through the upregulation the expression of tight junction proteins), decreased epithelial injury, and alterations in gut microbiota composition and metabolites (e.g., SCFAs and bile acids, as observed with Rh4) (156). By synchronizing the inhibition of these pathways, ginsenosides significantly diminish intestinal inflammation, lower oxidative stress, and ameliorate IBD.

### 5.4 Gut microbiota

Increasing evidence has revealed an association between the gut microbiota and IBD, where dysbiosis is either a cause or consequence of the development and progression of IBD. Several studies in DSS-induced colitis animal models have demonstrated that ginsenosides modulate the gut microbiota, enhancing beneficial microbial populations and mitigating colitis-related symptoms. DSS treatment reduces tryptophan metabolite levels by modulating gut microbiota composition. Ginsenosides CK and Rg1 improve the microbial balance, particularly by increasing the abundance of probiotic strains such as *Lactobacillus* and *Akkermansia* and enhancing the release of tryptophan metabolites. Rg1 and CK further regulate multiple microbial metabolic pathways, particularly those of tryptophan. Both CK and Rg1 mitigate IBD symptoms in mice by modulating the gut microbiota and increasing microbiota-derived tryptophan metabolites (159, 160). In addition, CK improves colitis by substantially reducing the abundance of *Bacteroides*. It improves disease outcomes, elevates tight-junction protein expression, and suppresses pro-inflammatory cytokine expression (TNF-α, IL-6, IL-1β, and IL-17). Fecal microbiome transplantation further confirms that its protective effects are primarily driven by changes in the gut microbiota (161). Korean Red Ginseng substantially improves colitis symptoms mice by promoting gut microbial diversity, increasing abundance of beneficial bacteria (e.g., *Lactobacillaceae* and *Prevotellaceae*), and suppressing the abundance of pathogenic taxa (e.g., Proteobacteria, *Tannerellaceae*, and *Oscillospiraceae*). It also minimizes epithelial injury and inflammation, enhances mucus production, and facilitates epithelial repair via β-catenin/TCF4 signaling (162). Similarly, ginsenoside Rb3 increases the relative abundance of *Lactobacillus* and decreases that of *Bacillus* in mice with colitis, thereby improving colitis symptoms. Beyond altering microbial diversity, it also substantially elevates the expression of tight junction proteins (Occludin and ZO-1) and reduces inflammatory cell infiltration and pro-inflammatory cytokine levels (TNF-α, IL-1β, IL-15, IL-17A, and IL-6) (163). In mice, ginsenoside Rg3 improves colitis symptoms by inhibiting the NLRP3 inflammasome activation and altering gut microbiota composition. Treatment with Rg3 enhances the relative abundance of *Prevotellaceae, Paraprevotella*, and *Chlamydia* but decreases the relative abundance of *Rikenellaceae* and *Clostridium sensu stricto 1*. These results suggest that Rg3 maintains a stable microbial balance in mice, likely by inhibiting the NLRP3 inflammasome activation (164). Additionally, in a TNBS-induced colitis rat model, red ginseng was found to promote beneficial *Lactobacillus* and diminish pathogenic *Escherichia coli* growth, contributing to the alleviation of experimental colitis symptoms (165).

## 6 Challenges and future directions

*In vitro* and *in vivo* studies have demonstrated the therapeutic potential of ginseng and its bioactive compounds in ameliorating the experimental colitis. Although numerous natural compounds, including curcumin, ginger, and resveratrol, have been shown to reduce oxidative stress, restore gut microbial equilibrium, and modulate inflammatory signaling pathways to treat IBD (166–168). Nonetheless, ginseng stands out due to its adaptogenic potential, unique structural diversity, and widespread biotransformation into bioactive metabolites by the gut microbiota, which may offer special therapeutic benefits. However, the application of ginsenosides has been hindered because of their low bioavailability, including factors such as poor water solubility, low permeability across biological membranes, gastrointestinal issues, susceptibility to enzymatic degradation, and total metabolism within the body, which contributes to a suboptimal absorption profile that has impeded the therapeutic possibilities of these promising compounds (169). These critical gaps must be addressed to enable translation from the bench to the bedside.

Future research should focus on developing advanced drug delivery systems (DDSs) to overcome the limitations of ginsenosides. Nanoparticle-based DDSs improve therapeutic potential by enhancing targeting, stability, and bioavailability, while reducing toxicity. Advances in nanotechnology have led to the development of multifunctional nano-agents capable of both passive and active targeting, demonstrating significant option for the treatment of IBD (170). For example, liposomes are versatile advanced nanocarriers that enhance drug delivery by increasing stability, improving bioavailability, enabling targeted delivery, and minimizing toxicity. Their unique structure enables the encapsulation of both hydrophilic and hydrophobic medications, rendering them promising drug delivery platform for IBD treatment (171, 172). Liposomal formulations such as Rg3-loaded liposomes greatly enhance pharmacokinetics and therapeutic potential, thereby addressing challenges such as low solubility (173). Hydrogels are biocompatible, stimulus-responsive 3D polymer networks that function as viable DDSs, particularly for the treatment of IBD. They facilitate precise, regulated drug delivery and enhance treatment outcomes. The bioavailability, retention time, and efficacy of ginsenosides (such as Rg3, Rh2, and CK) are greatly improved when incorporated into hydrogels. These hydrogels can adhere to inflamed colonic tissues, enhance drug absorption (e.g., by blocking P-glycoprotein), and support immune recovery, while eliminating pathogenic bacteria (174, 175). Overall, hydrogels as DDSs show considerable potential as favorable carriers of ginsenosides for the treatment of IBD. In order to facilitate their translation into clinical practice, future research should also concentrate on optimizing dose regimens, conducting well-designed clinical trials, and testing these DDSs in clinically relevant chronic colitis models. Furthermore, multi-omics approaches, such as metagenomics, transcriptomics, and metabolomics, could provide critical insights into the interactions among ginsenosides, gut microbiota, and host responses, underscoring the potential for microbiome-driven personalized treatment approaches in IBD (176, 177). For example, integrated metagenomics, metabolomics, and network analysis have been employed to detect alterations in gut flora, metabolic biomarkers, and host targets linked to the therapeutic benefits of ginsenosides in acetic acid induced experimental colitis rat model (178). Moreover, integrating natural compounds with anti-inflammatory and microbiota-regulating properties, such as ginsenosides, into combination therapies—alongside probiotics, prebiotics, or traditional medications—can yield synergistic effects and address existing challenges in IBD management.

Structure–activity relationship (SAR) studies have indicated that the quantity, variety, and position of sugar moieties in ginsenosides are critical determinants of their bioavailability, immune responses, and pharmacokinetic properties. Processing techniques, such as deglycosylation and steaming, yield rare ginsenosides that exhibit enhanced bioavailability and immunomodulatory effects. SAR insights indicate that targeted removal or retention of certain sugars (e.g., C-20 versus C-6), optimization of stereochemistry, reduction of molecular weight, and maintenance of essential functional groups improve receptor binding, membrane permeability, and metabolic stability (179, 180). For instance, intestinal microbial deglycosylation of ginsenoside Rb1 to Compound K improves its bioavailability and exert anti-inflammatory activity, highlighting the significance of the number and position of sugar moieties in influencing biological activity (181, 182). In general, the SAR-guided design converts ginsenosides into more potent and bioavailable immunomodulators, with significant promise for addressing conditions such as IBD.

Another significant challenge is the variability in ginsenoside composition and microbial transformation, which results in inconsistent outcomes and is due to the lack of standardization of ginseng products across species, processing techniques, dosages, and administration routes. The complex chemistry of ginseng makes it even more difficult to link its biological effects to specific compounds. Thus, the lack of standardized preparations remains a significant obstacle to reproducibility, clinical application, and regulatory approval (183, 184). Clinical translation of ginsenosides requires comprehensive PK/PD characterization, dose optimization, and safety assessment. Standardized formulations are crucial for ensuring consistent effectiveness and reproducible host–microbiome reactions. Future advancements depend on carefully crafted clinical trials and targeted strategies to facilitate effective therapeutic applications in conditions such as IBD.

## 7 Conclusion

Numerous *in vitro* and *in vivo* studies have demonstrated the potential of ginseng and its derivatives in the management of IBD. They have long been used as food ingredients and are well known for their healing effects in the treatment of various illnesses. Although current therapies have notably improved IBD management, their clinical application is still constrained by inconsistent treatment responses, reduced response over time, side effects, high costs, and issues regarding long-term safety, highlighting the continued necessity for innovative treatment strategies. Therefore, ginseng and its bioactive compounds may represent potential options for treating gastrointestinal disorders, as they help restore immune balance, diminish inflammation, alter gut microbiota and metabolism, suppress key inflammatory signaling pathways, and control the expression of pro-inflammatory cytokines and inflammatory mediators, which collectively enhance cellular differentiation and compensate for inflammatory injury in the colonic mucosa of experimental animal with IBD. Considering the persistent burden of symptoms such as diarrhea and abdominal pain in patients with IBD, ginsenosides may provide a promising therapeutic potential. Nonetheless, strong clinical evidence remains scarce, and well-designed randomized controlled trials, along with more stringent regulatory frameworks, are essential to confirm their therapeutic potential. Future directions for ginsenoside research should embrace a multidisciplinary translational approach that incorporates formulation science, microbiome studies, systems biology, and clinical research. Such a roadmap is imperative to thoroughly elucidate the therapeutic potential of ginseng and its derivatives and support their future clinical development for the treatment of IBD.
